# A cluster-randomized controlled trial to reduce sedentary behavior and promote physical activity and health of 8-9 year olds: The Transform-Us! Study

**DOI:** 10.1186/1471-2458-11-759

**Published:** 2011-10-04

**Authors:** Jo Salmon, Lauren Arundell, Clare Hume, Helen Brown, Kylie Hesketh, David W Dunstan, Robin M Daly, Natalie Pearson, Ester Cerin, Marj Moodie, Lauren Sheppard, Kylie Ball, Sarah Bagley, Mai Chin A Paw, David Crawford

**Affiliations:** 1 Centre for Physical Activity and Nutrition Research, Faculty of Health, Medicine, Nursing and Behavioral Sciences, Deakin University, Victoria, Australia; 2 Baker IDI Heart and Diabetes Institute, Melbourne, Victoria, Australia; 3 School of Sport, Exercise and Health Sciences, Loughborough University, UK; 4 Institute of Human Performance, The University of Hong Kong, Hong Kong, Hong Kong SAR; 5 Deakin Health Economics, Faculty of Health, Medicine, Nursing and Behavioral Sciences, Deakin University, Victoria, Australia; 6 Department of Public and Occupational Health, EMGO Institute for Health and Care Research, VU University Medical Center, Amsterdam, The Netherlands

## Abstract

**Background:**

Physical activity (PA) is associated with positive cardio-metabolic health and emerging evidence suggests sedentary behavior (SB) may be detrimental to children's health independent of PA. The primary aim of the Transform-Us! study is to determine whether an 18-month, behavioral and environmental intervention in the school and family settings results in higher levels of PA and lower rates of SB among 8-9 year old children compared with usual practice (post-intervention and 12-months follow-up). The secondary aims are to determine the independent and combined effects of PA and SB on children's cardio-metabolic health risk factors; identify the factors that mediate the success of the intervention; and determine whether the intervention is cost-effective.

**Methods/design:**

A four-arm cluster-randomized controlled trial (RCT) with a 2 × 2 factorial design, with schools as the unit of randomization. Twenty schools will be allocated to one of four intervention groups, sedentary behavior (SB-I), physical activity (PA-I), combined SB and PA (SB+PA-I) or current practice control (C), which will be evaluated among approximately 600 children aged 8-9 years in school year 3 living in Melbourne, Australia. All children in year 3 at intervention schools in 2010 (8-9 years) will receive the intervention over an 18-month period with a maintenance 'booster' delivered in 2012 and children at all schools will be invited to participate in the evaluation assessments. To maximize the sample and to capture new students arriving at intervention and control schools, recruitment will be on-going up to the post-intervention time point. Primary outcomes are time spent sitting and in PA assessed via accelerometers and inclinometers and survey.

**Discussion:**

To our knowledge, Transform-Us! is the first RCT to examine the effectiveness of intervention strategies for reducing children's overall sedentary time, promoting PA and optimizing health outcomes. The integration of consistent strategies and messages to children from teachers and parents in both school and family settings is a critical component of this study, and if shown to be effective, may have a significant impact on educational policies as well as on pedagogical and parenting practices.

**Trial registration:**

ACTRN12609000715279; Current Controlled Trials ISRCTN83725066

## Background

In the past few decades, rates of overweight and obesity and related metabolic and cardiovascular risk factors in children have steadily increased worldwide [1-4]. Physical activity plays an important role in the prevention of metabolic and cardiovascular health risk factors in children [5]. Increasingly, evidence suggests that sedentary behaviors, such as prolonged periods of television viewing, electronic games, and computer use (collectively called screen-time) may adversely affect children's weight status, independent of physical activity participation [6,7]. While these screen behaviors are most commonly performed during children's leisure-time there are many opportunities throughout the day for children to be sedentary (e.g., being driven to school, sitting in class, and sedentary homework). Further, while some longitudinal evidence suggests that increases in the time children spend in sedentary behaviors seem to be off-set by corresponding decreases in physical activity during the primary school years [8], other studies have reported independent changes in these behaviors over time suggesting that they should be viewed as separate rather than converse constructs [9].

There is emerging evidence that not just screen time, but total sedentary time may be detrimental to children's health. A cross-sectional study of 208 Portuguese children (mean age 9.8 years) found positive associations between accelerometer-measured sedentary time (defined as < 500 Actigraph counts per minute [cpm]) and insulin resistance, and inverse associations between moderate- to vigorous-intensity physical activity (MVPA; ≥2, 000 cpm) and insulin resistance [10]. A study of 111 US children (aged 3-8 years), however, found no cross-sectional associations between time spent sedentary (< 100 cpm) and systolic or diastolic blood pressure (BP) [11]. Nevertheless, that study reported that children in the highest tertile for proxy-reported television viewing time (approximately 155 mins/day) were significantly more likely to have higher systolic and diastolic BP compared with children in the lowest tertile (approximately 8 mins/day).

Observational evidence from studies among adults suggests that the manner in which time spent sedentary is accumulated may also be detrimental to health. For example, a cross-sectional study with 168 Australian adults (mean age 53 years) found that independent of MVPA levels, those with less frequent interruptions to accelerometer-measured sedentary time (≥100 cpm) with light-intensity physical activity had less favourable health profiles (waist circumference, body mass index, triglycerides, 2-hour plasma glucose) compared to those with more frequent interruptions [12]. Interestingly, the average duration of light-intensity breaks was less than five minutes, suggesting that even brief interruptions to time spent sedentary may be beneficial to health. To our knowledge, no observational or experimental studies have examined the association of interruptions to sedentary or sitting time and health in children, nor has the role of light-intensity physical activity and children's health been previously studied.

While few intervention studies have examined the effectiveness of strategies to reduce children's overall sedentary time, several review papers have summarized the effectiveness of interventions to reduce children's screen time [6,13,14]. While this evidence suggests these strategies (delivered primarily through school-based curriculum), have positive effects on children's weight and have successfully reduced TV viewing, as noted earlier, there are many opportunities to be active throughout the day both at school and at home [15] and few if any of these interventions to reduce children's screen time have resulted in corresponding increases in physical activity.

Several studies have reported significant positive effects on children's physical activity in the school setting by targeting the school curriculum or through changes in the school environment [16-19]; however, few studies report on intervention effects on children's sedentary time. A recent experimental study by Benden et al. among children in four classes in Central Texas introduced standing desks into classrooms and found that after 12 weeks all children were standing for 75% of the time [17]. However, the intervention only targeted energy expenditure at school and did not incorporate strategies to increase energy expenditure or reduce sedentary behavior outside of school hours. A further challenge with this type of intervention is whether the aim is to reduce children's sedentary time, increase physical activity or both. In a meta-analysis of intervention studies that aimed to promote young people's physical activity or reduce screen time, pooled effect sizes of 0.12 and -0.29 respectively were reported [14]. The authors concluded that strategies to reduce sedentary behavior appeared to be more effective than strategies to increase physical activity. However, the efficacy of strategies to increase physical activity and reduce sedentary behavior separately and in combination has not been examined.

Ecological models suggest that settings-based approaches may be an effective method for intervening with children's health behaviors [20,21]. Interventions that target places and contexts in which large numbers of children are sedentary or active are likely to have a greater public health impact than approaches that involve one-on-one program delivery. In addition, an important aspect in the development of effective and efficacious behavioral interventions is the use of a theoretical framework [22,23]. The use of behavioral theory helps guide the development of strategies that are most likely to result in changes in behavior through targeting the key mechanisms or mediating constructs of change [24,25]. Commonly employed theories in children's physical activity and sedentary behavior intervention studies include: social cognitive theory [26]; theory of planned behavior [27]; and behavioral choice theory [28]. A limitation of many of these theories is the focus on intrapersonal factors, which on the one hand are important for targeting change at the individual level, but are less useful when targeting changes at the population level. More recently, but less frequently, ecological models such as the social ecological model of health promotion [29] and the family-based ecological systems theory [20] have also been employed in interventions to promote children's physical activity with mixed success [30,31].

Very few studies, even those that report use of behavioral theory in the design of their intervention, examine the mediators or mechanisms of behavior change. Several reviews of mediators of physical activity interventions in children and youth have identified key mediators to target including: self-efficacy; behavioral capability; perceived social support; physical activity knowledge and beliefs; and enjoyment of or preference for physical activity [32-34]. Just two studies have examined possible mediators of change in sedentary behaviors such as television viewing and computer use in young people [35,36]. The DOiT study was an obesity prevention intervention based on the theory of planned behavior and habit strength theory [37] that aimed to improve dietary and physical activity habits as well as reduce sedentary time of Dutch adolescents [35]. In that study there were no mediating effects of attitude, subjective norms (i.e. the degree to which an individual is inclined to agree with the expectation of other important persons' opinions, normative beliefs), behavioral control or habit strength on youth screen time. Based on the self-determination theory [38] and the theory of meanings of behavior [39], the Get Moving! program was a media-based intervention delivered via the school setting that aimed to increase physical activity and decrease sedentary behaviors in predominantly Latino middle school girls in California, USA [36]. The authors found a non-significant trend for a mediating effect of intrinsic motivation to be physically active on television viewing time. No other mediating effects were observed.

It is therefore important that intervention studies not only target key mediators that lie on the behavior change pathway, but that these pathways are then tested statistically. This will ensure a better understanding of why an intervention worked or not and will further inform the utility of behavior change theories. Another often-overlooked aspect of children's health behavior change interventions is the economic cost of program delivery. Not only is it important to test whether an intervention works and why, it is also critical that it is cost effective. Cost-effectiveness analysis combines effectiveness and cost data to show whether an intervention represents 'value for money', with results expressed as incremental cost-effectiveness ratios. A range of standard methods are available to guide economic evaluation of an intervention program [40,41]. For example, the Assessing Cost Effectiveness (ACE)-Obesity study examined the economic evaluation of thirteen interventions which targeted unhealthy weight gain in children and adolescents [42,43]. While the cost-effectiveness of interventions varied greatly, the most cost-effective strategies included 'Reduction of TV advertising of high fat and/or high sugar foods and drinks to children', 'Laparoscopic adjustable gastric banding' and the 'multi-faceted school-based programme with an active physical education component'[42]. Further research is required to identify the cost-effectiveness of strategies to reduce children's sedentary behavior and promote physical activity in the school and home settings.

This proposal builds on our program of research [42,44,45] aimed at identifying effective and cost-effective strategies that positively influence children's health behavior and translate to improved health outcomes. This paper presents a summary of the Transform-Us! intervention including its aims, development, intervention methods and assessment protocols.

### Aims

The primary aim of the Transform-Us! study is to determine whether an 18-month, behavioral and environmental intervention in the school and family settings results in higher levels of physical activity and lower rates of sedentary behavior among 8-9 year old children compared with usual practice (post-intervention and 12-months follow-up). The secondary aims are to determine the independent and combined effects of PA and SB on children's cardio-metabolic health risk factors; identify the factors that mediate the success of the intervention; and determine whether the intervention is cost-effective.

### Study Protocol Overview

Transform-Us! is a four-arm cluster randomized controlled trial with primary schools in Melbourne, Australia being the unit of randomization. The intervention will run for approximately 18 months (end of Term 2, 2010 to end of Term 4, 2011), with a 12-month tapered maintenance period in 2012. Transform-Us! is funded by a National Health and Medical Research Council Grant (No.533815). Ethical approval was obtained from the Deakin University Human Research Ethics Committee (EC 141-2009), the Victorian Department of Education and Early Childhood Development (2009_000344) and the Catholic Education Office (Project Number 1545).

## Methods/Design

### Study population

Twenty primary schools within a 50 km radius of Melbourne will be involved in Transform-Us!. All year 3 children in the intervention schools will receive the program and all year 3 children in all schools will be invited to participate in the evaluation of the intervention with the aim to recruit 600 students. Due to significant increases in sedentary behaviors and declines in physical activity among children in primary school, 8-9 year olds were considered a key target population. In addition, for practical purposes a three-year study implemented in year 3 would ensure children remained at primary school throughout the entire study ensuring ease of follow-up.

### Recruitment of schools

School principals will be contacted via fax or email and invited to participate in the Transform-Us! study. All interested principals (and teachers if in attendance) will be given a detailed presentation outlining the program and required commitment. Principals who agree to participate in the study will then be provided with a plain language statement and consent form to be signed and returned prior to participation. As the Transform-Us! program will involve modification to the delivery of the school curriculum, approval from the school council/board will also be required.

### Recruitment of participants

All year 3 children in eligible schools will be provided with an information pack for their parents or carers/guardians (hereafter referred to as parents) containing a plain language statement and consent form for the parents' and child's participation. As the school will have consented to the program being delivered to all year 3 children and parents, consent will only be required for the evaluation components. Parents will be able to nominate which assessment components they give consent for their child to participate in (i.e., accelerometer, inclinometer, anthropometry, survey, blood pressure and/or blood sample, or all components). At baseline, all year 3 teachers will be provided with an information pack containing a plain language statement and consent form to be signed and returned prior to participation in the evaluation assessments. To maximize the sample and to capture new students arriving at intervention schools, recruitment will be on-going up to the post-intervention time point. The schools, teachers and participants will not be paid to participate in Transform-Us!.

### Sample size calculations

It is expected that the intervention effects on behavioral and biological primary outcomes will be moderate in size (standardized difference ~ 0.32, equivalent to a mean change in objectively-measured PA of 8 minutes per day [SD = 18 min] and a change in body mass index (BMI) units (age-sex difference from population norm data) of 1.9 kg/m^2 ^[SD = 0.25]) [25,46]. Without accounting for school cluster effects, the number of participants needed to detect a standardized difference of 0.32 with 0.8 power for sedentary behavior (SB-I) and physical activity (PA-I) alone and in combination (SB+PA-I), using a significance level of 0.05 with a two-tailed test, assuming a retention rate of 91% (based on the team's previous experience) and 20 participating schools, would be 340 in total (17 per school). With a previously observed school clustering effect of 0.018 (intra-class correlations [ICCs] for sedentary behavior, physical activity and BMI outcomes estimated using data from a previous intervention study)[44], the minimal total number of participants needed is 520, i.e., 26 per school. Hence, we will conservatively recruit 30 participants per school, giving a total sample size of 600 participants.

Moderate mediated effects of the intervention on the behavioral outcomes are expected (here a moderate mediated effect size is defined as standardized regression coefficients α and β of ~ 0.39)[47]. According to a 2007 simulation study by Fritz and MacKinnon [48], to detect a moderate mediated effect size with 0.8 power, using a significance level of 0.05 with a two-tailed test and bias-corrected bootstrap methods, ~ 61 independent observations are needed. If we assume a 9% rate of loss to follow-up, an average school cluster effect of 0.03 for the mediators (ICCs estimated using data from a previous intervention study)[44] and an average of 30 observations per school (see above), the sample size needed to detect a moderate mediated effect size would be 100 (20 per school). Hence, the sample size needed to detect moderate intervention effect sizes with 0.8 power for the primary outcomes (n = 600) will also be sufficient to detect a moderate-sized effect with 0.8 power for the mediating variables.

### Randomization

A listing of Melbourne suburban primary schools (n = 367), their enrolment number and associated suburb socioeconomic index for areas (SEIFA) score (suburb disadvantage score) will be obtained. Schools with an enrolment of over 300 students will be grouped in quintiles of SEIFA score and schools from the first, third and fifth quintiles will be selected to represent low, mid and high SEIFA strata respectively. Schools in each stratum will be randomly ordered with probabilistic weighting according to enrolment number, and will be approached consecutively and offered participation. Schools will then be randomly allocated to either SB-I, PA-I, SB+PA-I or current practice control (C).

## Intervention

### Development and Pilot Phase

A previous research-to-practice study (Switch-2-Activity) demonstrated the feasibility of teachers delivering materials targeting the promotion of children's physical activity and reductions in screen time [49]. For the current study, seven teachers, including a vice-principal, were interviewed regarding the feasibility of introducing regular classroom standing breaks. Short breaks were considered feasible with consistent feedback regarding the duration; anything longer than 2-minutes was viewed as being unlikely to be adopted by teachers. A subsequent pilot study to assess the feasibility and effectiveness of regular 2-minute classroom breaks found a 20-minute decline in children's objectively measured sedentary time during class and a corresponding 20-minute increase in moderate- to vigorous-intensity physical activity [50]. All class-based components of Transform-Us! were developed by the investigators with input from current and previous primary schools and representatives of the Victorian Department of Education and Early Childhood Development.

### Theoretical basis of Transform-Us!

Physical activity interventions that base their strategies on behavioral theories are more likely to be effective than atheoretical approaches [51]. Table 1 shows the mediators that will be targeted in Transform-Us!. These mediators are based on elements of the behavioral theories that have previously been shown to be effective in encouraging behavior change in children's physical activity and sedentary behavior [44,51-54] including: social cognitive theory [26]; behavioral choice theory [28] and ecological systems theory [20]. These theories recognize that there are multiple levels of influence on health behavior including intrapersonal (e.g., awareness, self-efficacy, enjoyment), interpersonal (e.g., parents, siblings, peers, teachers), physical environmental (e.g., television in child's bedroom, access to parks/playgrounds), and policy influences (e.g., school physical activity policies and timetables). As previous research has shown consistent sex differences in physical activity [55] and in some sedentary behaviors (particularly computer use and playing electronic games [56-58]) and sex was a significant moderator in the Switch-Play study [44] the intervention will be tailored for boys and girls wherever possible. See Table 2 for a summary of the intervention strategies.

**Table 1:** Theoretical* basis of the Transform-Us! intervention and links to program objectives

Constructs	Mediators or determinants	Program Objectives
Intrapersonal		
Confidence	Self-efficacy	Improve confidence in ability to be active or reduce sedentary time
Preference	Enjoyment	Increase enjoyment and preference for physical activity
Expectations	Benefits/barriers	Increase knowledge of benefits & strategies to overcome barriers
Expectancies	Evaluation of anticipated outcome	Alter perception of pros and cons of being more active
Skills	Self-management	Self-rewards, self instructions, TV viewing styles
Behavioral rehearsal	Self-monitoring & contracting	Goal setting, contracting with others, rewards
Interpersonal		
Observational learning	Modelling by parents/siblings	Encourage parents & siblings to reduce their own SB & increase PA
Social support	Modelling/social support	Encourage parents & siblings to support child to spend less time in SB & more time in PA; teachers encourage/support PA during recess/lunch
Social structure	Rules	Parents enforce rules regarding limiting screen time at home, during meals, during daylight hours
Environmental		
Imposed environment	Availability	Increase the amount of PA equipment available at school & home. Reduce the availability of TVs/computers/electronic games at home
Imposed environment	Access	Increase access/opportunities for PA at school & at home. Decrease access to TV/computers/electronic games at home
Imposed environment	Policy	Interrupted sitting during class-time; presence of supervising teachers during recess/lunch

* Based on social cognitive theory [26], behavioral choice theory [28], ecological systems theory [20]

**Table 2:** Transform-Us! intervention components

	SB-I	PA-I	SB+PA-I
**School setting**			

Curriculum component	• 18 key learning messages (9 per year)	• 18 key learning messages (9 per year)	• 18 key learning messages (9 per year)
Class strategies	• Standing lessons (1 × 30-min/day) • Active 2- min breaks after 30-min class time	NA	• Standing lessons (1 × 30-min/day) • Active 2- min breaks after 30-min class time
Physical environment	• Standing easels • Novelty timer	• Provision of sporting equipment, line markings and signage • Provision of pedometers	• Standing easels • Provision of sporting equipment, line markings and signage • Provision of pedometers

**Family setting**			

Homework tasks	• Reduce sitting time while completing home work	• Homework tasks incorporate PA	• Homework tasks incorporate PA and reductions in sitting time
Newsletters	• Tips for reducing sitting time at home	• Tips for increasing PA at home	• Tips to reduce sitting time and promote PA at home

PA: physical activity; SB: sedentary behavior; PA-I: physical activity intervention; SB-I: sedentary behavior intervention

### Sedentary behavior intervention arm (SB-I)

Reducing uninterrupted time spent sitting during school hours will be aimed for in the school setting; and reducing overall sitting time and discretionary screen-based behaviors (i.e., television viewing, computer use and electronic games) will be aimed for in the family setting. In addition, key mediators of sedentary behavior change will be targeted (Table 1).

### School setting

#### Curriculum-based key learning messages

Key learning messages will be adapted from Switch-Play materials incorporating key principles of behavior change and delivered by classroom teachers in 18 lessons (9 lessons per year). Teachers will be provided with complete lesson plans but encouraged to modify the materials to suit their class and teaching style. The second year of the intervention will reinforce and enhance the lessons from the first year. Key messages will focus on raising awareness; self-monitoring; goal setting; behavioral contracts; social support (team-based activities at school; homework to do with parents); and feedback and reinforcement (external and intrinsic rewards). All lessons are developed in line with the Victorian Essential Learning Standards for year levels 3 and 4. Furthermore, children will be encouraged to meet the national recommendations for young people of less than two hours per day in electronic entertainment media [59].

#### Interrupting classroom sitting time

Teachers will modify the delivery of one class lesson per day (~30 minutes) so that children will complete the lesson standing up. Teachers will be provided with a suite of standing lesson delivery methods that can be modified to any class topic (e.g., 'Stations around the room' shown in Appendix 1). If administered as intended, this should result in approximately 150-minutes less sitting time per week. In addition, every two-hour classroom teaching block will be interrupted every 30 minutes with a 2-minute guided light-intensity activity break (e.g., 'Bean-bag Spelling/counting' shown in Appendix 2). This will equate to a total of six minutes interrupted sitting time every two hours. On average, schools in Victoria have two 2-hour teaching blocks per day so this should result in up to 60-minutes less sitting time per week. Teachers will be provided with a menu of activities to deliver during the 2-minute breaks.

#### Environmental cues and prompts

Each class will be provided with several standing easels so that children can rotate learning activities at 'standing stations'. In addition, a novelty timer will be given to each class so that teachers can monitor 2-minute standing breaks and every 30-minutes of sitting class time.

### Family setting

#### Newsletters

Nine newsletters per year (18 in total) will be sent home to parents providing project updates and tips on reducing their child's sedentary behaviors. The newsletters will support the key learning messages delivered to the children in the classroom and will help parents reinforce maintaining children's screen-time to a minimum. They will be translated into the most common languages spoken among the families with non-English speaking backgrounds. Newsletters will incorporate family based activities for parents to complete with their child and contain information about ways to reduce their child's screen time, including the effective use of rules (i.e., no television viewing during mealtimes, restrictions on small screen use, etc.).

#### Homework assignments

Homework tasks will be modified to reduce sitting time while completing them at home (e.g., complete worksheets while standing at the kitchen bench). Children will also be given homework tasks to complete with their parents; for example, to switch off the television for a whole weekend day and do something with their parents (a menu of alternative light-intensity activities will be provided).

### Physical activity intervention arm (PA-I)

Increasing or maintaining moderate- to vigorous-intensity physical activity (e.g., active play, organized and non-organized games) during recess and lunch breaks will be targeted in the school setting and time spent outdoors will be targeted in the family setting. The key mediators of change in physical activity will also be targeted (shown in Table 1).

### School setting

#### Curriculum-based program

As for SB-I, 18 key learning messages (9 lessons per year) modified from Switch-Play [44] but focusing on increasing physical activity will be delivered by teachers. Children will be encouraged to meet the national physical activity recommendations for young people of 60-minutes of moderate- to vigorous-intensity physical activity every day. All lessons will be developed in line with the Victorian Essential Learning Standards for year levels 3 and 4.

#### Physical activity during recess and lunch breaks

Physical activity will be promoted and encouraged during recess and lunch breaks. Based on previously efficacious intervention strategies promoting physical activity during recess and lunch breaks [60], schools will be provided with sports equipment to make available for children to use in recess and lunch breaks, and teachers and peers will provide encouragement and support for active games.

#### Environmental cues and prompts

Signage will be used to promote physical activity in schools and will be regularly updated. Consistent with previously successful interventions [61], schools will select and receive novel line markings promoting active play in asphalt areas in the school playground. Each class will be provided with a set of pedometers for use during the key learning messages based on physical activity capability. Furthermore, the pedometers can be rotated throughout the class for children to gain awareness of their steps in different environments (e.g. weekday, weekend, school camp).

### Family setting

#### Newsletters

Eighteen newsletters (nine per year) will be sent home to parents providing project updates and tips on promoting their child's physical activity. The newsletters will support the key learning messages delivered to the children in the classroom and will help parents reinforce physical activity promotion at home. They will be translated into the most common languages spoken among the families with non-English speaking backgrounds. Newsletters will incorporate family-based activities for parents to complete with their child. For example, newsletters will contain information about activities to do at home and in their neighborhood (suitable for boys and girls). Parents will also be directed to the Kinect Australia website http://www.kinectaustralia.org.au/content/Public/Homepage.aspx and free Infoline, both of which contain information for parents on ways to engage their child in physical activity at home, ways to be active with their child, as well as identifying specific places in their own neighborhood where they can take their child to play (e.g., playgrounds, walking trails, local sports clubs).

#### Homework assignments

Homework tasks will be modified to incorporate physical activity and children will be encouraged to complete these tasks with their parents (e.g., go for a walk with their parents and write about where they went and what they saw; mathematics homework using their stride as the unit of measurement).

### Combined sedentary behavior and physical activity intervention arm (SB+PA-I)

Schools randomized to the combined SB+PA-I intervention arm will receive a blended version of the two interventions, but with the same intervention 'dose'. For example, when children in this arm complete a behavioral contract to switch off the television, they will be encouraged to participate in physical activity (SB-I children will not be directly encouraged to participate in activity when they switch off their television). The combined intervention arm will include 18 class lessons (9 per year), standing lessons and interruptions to children's classroom sitting time (short breaks), the promotion of physical activity during recess and lunch breaks, and 18 newsletters to parents.

### Control - usual practice

Schools assigned to the usual practice control group will be asked to continue their usual lesson delivery and will receive all intervention materials (apart from line markings) at the completion of the 12-month follow-up period

### Teacher training

In the first year of the intervention (2010), all participating year 3 teachers at intervention schools will be required to attend a professional development (PD) session. In the second year of the intervention (2011), all year 4 teachers will undergo the same training. There may be some teachers who teach both years of the intervention, or the second year may contain new teachers who have not been previously involved in the delivery of the intervention. The PD session will run for approximately two hours and will inform and/or refresh the teachers about the intervention including the aims, procedures of the study and strategies to be used. A mid-year morning tea meeting will be held with teachers to ensure that they are delivering the intervention as intended and to answer any questions or solve any difficulties they may be experiencing in intervention delivery. At the beginning of the third year (2012), all year 5 teachers at the intervention schools will be trained in their specific intervention components but will have no further contact or support from the study team throughout the year. This tapered approach will enable the maintenance of the strategies to be examined.

### Measurement protocol

Assessments will be conducted at baseline (Feb-May 2010), mid-intervention (Nov-Dec 2010), post-intervention (Nov-Dec 2011), and 12-months follow-up (Nov-Dec 2012). To minimize participant burden, blood pressure assessments will be taken at baseline, post-intervention and 12-months follow-up only, and blood samples will be taken at baseline and post-intervention only. All of the children's measurements (except the blood samples) will be conducted at school by trained research staff using regularly calibrated equipment. Children will complete the survey in small groups with trained research staff. The blood sample will be undertaken at a commercial pathology laboratory by a trained phlebotomist and the parent survey will be sent home to parents to complete.

### Primary outcomes

#### Sedentary time and physical activity

##### ActiGraph accelerometer

Sedentary time and physical activity will be objectively-assessed using the uniaxial function in the ActiGraph Model GT3X accelerometers http://www.theactigraph.com/. Children will wear the ActiGraph on a belt positioned over the right hip during waking hours (apart from during water-based activities) for eight days at each of the measurement points. Data will be collected in 15-second epochs. The movement count threshold for sedentary time will be set at 25 counts per 15-second epoch [62], and the number of breaks or interruptions to time spent sedentary will be defined as the frequency of occasions data exceeded 100 counts.min-1. The Freedson age-adjusted equation [63], will be applied to calculate the time spent in light (1.7-3.9 METS), moderate- (4.0-5.9 METs) and vigorous-intensity (≥6.0 METs) physical activity. We will also extract accelerometry data from specific times of the day (e.g., after-school hours, during class time) to identify when changes in sedentary time or physical activity may have occurred.

##### *activ*PAL™ inclinometer

A sub-sample of randomly selected children will concurrently wear a PAL Technologies Ltd, Glasgow, Scotland http://www.paltech.plus.com/products.htm inclinometer *activ*PAL™. The *activ*PAL™ is a small device (5 × 3.5 × 0.7 cm) weighing 20 g and is worn on a garter belt positioned at the mid-anterior aspect of the right thigh during waking hours (apart from water-based activities) for eight days at each of the measurement points. The following parameters will be calculated from this device: sedentary time (sitting/lying time); stand time (not walking); walk time; and sit-to-stand events. The device has been found to have acceptable validity in assessing these parameters in adults [64,65].

ActiGraph and *activ*PAL™ data validity criteria will be assessed prior to inclusion in analysis. A minimum of four valid days, including one weekend day will be required. To be included in analyses, a day will be considered valid if the child has at least eight hours of wear time per day or at least 50% wear time within periods of the day (e.g., classtime). Further, 20 minutes of consecutive zeros will be considered non-wear time. Recordings of over 16000 counts per minute (cpm) will be excluded as it indicates unit malfunction.

##### Survey measures

Behavioral information on the types of physical activities and sedentary behaviors in which children participate (not detectable by accelerometry or inclinometers) will be collected by a parental proxy-report version of the validated CLASS questionnaire [66]. Time spent outdoors will be assessed using a previously validated proxy-report measure [67].

### Secondary outcomes

#### Anthropometry: height, weight and waist circumference

Height will be measured to the nearest 0.1 cm using SECA portable stadiometers (mod 220). Weight will be measured to the nearest 0.1 kg using portable electronic Wedderburn Tanita scales. Two height and weight measurements will be taken and the average calculated. Where a discrepancy of over 1 cm or 1 kg is noted, a third measurement will be taken.

BMI (kg/m2) will be calculated and converted as recommended for analysis of longitudinal adiposity data [68]. This involves subtracting the sex-age population median (based on US data)[69] from the child's raw BMI score. Children will also be categorized as healthy weight or overweight/obese based on International Obesity Task Force definitions [70].

Waist circumference will be measured using a flexible steel tape at the narrowest point between the bottom rib and the iliac crest, in the midaxillary plane. If there is no obvious narrowing the mid-point between these two landmarks will be used [71]. Two waist circumference measurements will be taken and the average calculated. Where a discrepancy of over 1 cm is noted, a third measurement will be taken. Continuous data will be compared to UK charts [72]. Sex and age-specific waist circumference thresholds for children that correspond to clustering of cardiovascular disease risk factors will also be applied [71].

#### Blood pressure

After a quiet two-minute seated rest, children will have their blood pressure measured on their right arm using the OMRON HEM-907 automatic digital blood pressure machine. Three measurements will be taken one-minute apart on two occasions (one week apart). The first measurement on each occasion will be discarded. The remaining four measurements will be averaged to obtain systolic and diastolic blood pressure measurements.

#### Serum Biomarkers

A fasted, morning blood sample (16.5 ml) will be taken by a phlebotomist at a commercial pathology laboratory to assess children's cholesterol, high density lipoprotein (HDL) cholesterol, low density lipoprotein (LDL) cholesterol, triglycerides, glucose, insulin, C-reactive protein and 25-Hydroxy Vitamin D levels. Biomarkers will be taken at baseline and post-intervention data collection points only.

#### Survey measures

##### Mediators

Intrapersonal, interpersonal and environmental mediators of children's sedentary behaviors and physical activity will be assessed by parental proxy-report and child self-report survey (Table 1). Intrapersonal mediators include: self-efficacy; enjoyment and preference; sports competency; TV viewing style (e.g., channel surfing, selective viewing); benefits and barriers; and beliefs. Interpersonal mediators include: social norms; rules; social support in the neighborhood, at school, and at home; and parental modelling. Environmental mediators include: perceptions of the school environment; access and availability; and policy. These measures have been previously developed and have acceptable psychometric properties and predictive validity [52,56-58,73-78].

##### Moderators

Although not powered to conduct moderating analyses, exploratory analyses will examine moderating effects of sex of the child, parental country of birth, and parental education attainment.

##### Confounders

###### Sociodemographic characteristics

Child's age and sex, family structure (e.g., siblings, number people living in household), parents' age, sex, marital status, education attainment, postcode, and country of birth will be collected in the parent survey. Children will report the number of working cars their family has, the number of holidays they went on in the last year and if they have their own bedroom to assess sociodemographic position [79].

###### General health and family history

The responding parent will report the general health status of their child, and the medical history and family risk factors for diabetes and cardiovascular disease risk factors and their own height and weight.

###### Nutritional intake

Parent proxy-report food/drink frequency questions derived from food items previously identified from the National Nutrition Survey for the target age groups (8-12 years) as important contributors to energy and fat intakes, and thus the energy density of the diet, will be included (items include sweet and savoury snacks and high energy drinks) [80]. Literature supports the accuracy of parent reports of usual food intakes [81].

###### Stage of pubertal maturation

Since all children will be aged 8-9 years old at baseline, it is expected that they will all be pre-pubertal and thus the assessment of pubertal status is not considered necessary. However, to account for any potential growth (maturity) effects, growth rates (change in height) will be assessed from the height measurements taken at each assessment time point.

### Economic Evaluation

An economic evaluation will be undertaken to determine whether Transform-Us! is cost-effective. A societal perspective will be adopted, such that all costs and outcomes will be identified and valued regardless of who bears the cost, receives the benefits or provides the resources [82,83]. Three methods for collecting resource use data will be applied: a diary will be given to teachers to capture any extra preparation time or equipment needed to implement the intervention strategies; an item on the parent survey will capture how much time family members participate in completing school homework tasks; and project monitoring processes will be used to capture other resources used that are pertinent to implementation. Diaries will be completed each day for four weeks throughout the intervention period and at follow-up.

### Process Evaluation

Throughout the intervention (2010 and 2011), the project team will maintain regular contact with teachers. We will monitor the intervention delivery quantity (how many children participated in the various lessons) and quality (that the intervention was delivered as it was intended and any modifications to provided materials) throughout the intervention (teachers to record and rate lessons delivered, sections of class lessons completed with children, number of children present, engagement of the children). In addition to annual interviews with key personnel in the school (e.g., teachers, co-ordinators), we will make unannounced visits to schools to ensure that the teachers understand the intervention delivery requirements, to solve any difficulties or concerns, and to ensure that they are delivering the intervention as intended.

Subjective evaluations of intervention components will also be provided by children, parents and teachers throughout the intervention. At the mid- and post-intervention assessments, teachers will be asked to report on the lesson delivery (e.g., did you deliver the key learning messages, ease with which they were embedded in current curriculum, ease of delivery) and school-based strategy implementation (e.g., were you successful in getting students to complete standing lessons? Was the sporting equipment made available at recess/lunchtimes?). At the post-intervention assessments, teachers who were involved in year one, but not year two, of the intervention will be asked about their continuation of any of the strategies. At the 12-month follow-up assessments, all teachers who have been involved in the SB-I or SB+PA-I intervention in year one and/or year two will be asked to complete a survey to examine maintenance of the class based strategies (i.e., are you still conducting standing lessons?). The uptake of the intervention by teachers not previously involved in year one or two will also be determined.

At the mid- and post-intervention assessments, all participating children will be asked to describe what they know about Transform-Us!. Children in the three intervention groups will be asked to rate how they feel about the school-based components specific to their intervention group (e.g., standing while completing work, availability of sports equipment at recess/lunchtimes); and the home-based components specific to their intervention group (e.g., standing homework tasks, active family pastimes). At the mid- and post-intervention assessments, parents will be asked about the newsletters (e.g., number received, usefulness and their use of strategies to change their child's behavior contained within the newsletters).

### Data analysis

All statistical analyses will be performed using Stata 9.1 for Windows (StataCorp LP) and will adjust for clustering by observations by school (the unit of randomization). All analyses will be conducted using the intention to treat principle. Generalized Estimating Equations (GEE) [84] will be used to fit regression models to describe the effects of the intervention on key outcome variables among children. Separate models will be fitted to determine differences in key outcome variables in the intervention and control groups.

Separate models will be evaluated for each assessment point (mid-intervention; post-intervention; 12 months follow-up), adjusting for baseline levels of the outcome, and, where appropriate, baseline levels of the mediating variables. PA-I and SB-I intervention conditions will be the predictors of primary interest (entered in the form of two indicator variables and their interaction term). Analyses will be performed excluding and including children who were recruited to the study after baseline. For physical activity outcomes, we will compare the PA-I and SB+PA-I versus the SB-I and C groups. For sedentary behavior outcomes, we will compare SB-I and SB+PA-I versus the PA-I and C groups. For health outcomes, we will compare C versus the other three groups and the SB+PA-I versus the single intervention groups. All models will be adjusted for measured confounders. As it has been argued that adjustment for multiple comparisons should not be used when assessing evidence about specific hypotheses no adjustment for multiple comparisons will be performed [85]. To evaluate the magnitude and direction of the intervention effects mediated by intrapersonal, interpersonal and environmental processes, the product-of-coefficient method will be used [86]. A first set of GEE regression models will assess the impact of the intervention condition (regression coefficient α) on the residualized change score of the hypothesized mediators (Table 1), after controlling for significant covariates. A second set of GEE regression models will estimate the independent effects of the intervention condition and residualized changes in the mediators (regression coefficient β) on changes in the outcome variables. The significance of the product of the regression coefficients α and β (representing the mediated effect of the intervention) will be tested using bias-corrected bootstrap methods as outlined by Pituch and colleagues [87].

For the economic evaluation, pathway analysis will be used to identify component activities of the intervention in order to ascertain the associated resource utilization. The cost-effectiveness analysis will measure differential costs between the three intervention groups and C in relation to the outcomes, and intervention costs will be assessed as additional expenditure (savings) against C, expressed as an incremental cost-effectiveness ratio. Incremental cost-effectiveness ratios will be calculated as the cost AUD per BMI unit 'saved' and Disability-Adjusted Life Years (DALYs) 'saved'. The intervention will be modelled for one year as if applied to the Victorian population of children in the target group. The time horizon for measuring the associated health-care cost-offsets and DALY benefits will be the rest of life or 100 years. The reference year will be 2010. The interventions will be run through a model, developed as a part of the ACE-Obesity study, which converts BMI changes to DALY benefits and cost-offsets saved over the lifetime of the cohort [88]. The ACE-Obesity methodology will guide the evaluation to allow cost-effectiveness results to be directly comparable to those of the 13 interventions previously evaluated [89].

## Discussion

Sedentary behavior and physical activity may have independent detrimental and positive effects (respectively) on children's cardio-metabolic health. Very few interventions have targeted reductions in children's total sitting time in the school and family settings. Even fewer interventions have examined the separate and combined effects of targeting reductions in sedentary behavior and increases in physical activity and only two have used objective measures. This 18-month intervention with 12-month tapered maintenance period is unique in its approach to delivery of school curriculum and homework in less sedentary and more active ways. As the key agents of change in children's health behaviors, involvement of teachers and parents is critical. If shown to be successful and cost-effective this intervention may have implications for educational policy and practice, and ultimately the health of young children in Australia and elsewhere.

## Competing interests

The authors declare that they have no competing interests.

## Authors' contributions

JS took the lead in designing the study subsequently funded by a National Health and Medical Research Council Project Grant and in writing this manuscript.

LA contributed to drafts of this manuscript and coordinated comments from co-authors.

MM and LS wrote the sections related to the economic evaluation protocol.

CH, KH, RD, DD, EC, NP, HB, KB, DC provided expert input and support overall for the writing of this manuscript with particular emphasis on design, measures of physical activity and statistical analyses.

All authors read and approved the final manuscript.

## Appendix 1

'Road trip/Stations around the room' standing lesson strategy

### Aim

To complete small group activities related to a specific area of study, by physically moving between stations located around the room.

### Outline

The lesson is planned as a series of activities, placed at stations around the classroom. Children are organised into groups (allocated by the teacher). Groups spend a designated period of time at each station completing the activity, before moving onto the next station. All children must remain standing at each station as activities are designed to be easily performed standing (e.g. through use of high benches or clipboards; by keeping written work relatively minimal; by telling children to take turns at being 'scribe' etc).

Teachers can specify the group structure (number of children, how groups are selected), the number of stations, amount of time spent at each station and the associated activities.

### Examples

- Fill in worksheets

- Leave your group's answer on a pile at the station

*- Keep a journal of all of the activities *(write sentences or draw pictures)

- Write responses on large sheets of paper/whiteboards

### Suggestions for stations

**• English**: One chapter/event from a story at each station including comprehension questions.

**• L.O.T.E**.: Match up word-cards and picture-cards; sentences on large posters or interactive whiteboards with blank spaces for children to fill with the correct word (offer lists of choices).

Culturally relevant creative activities, such as origami.

**• Maths: **Set of addition, multiplication or simple division problems - one series per station.

Spatial tasks - fitting shapes into defined spaces (puzzles).

Problem-solving activities +/- problems- Example: "You have a bucket with 150 mL of water, a 40 mL jar and a 50 mL jar. How will you use these to measure out 30 mL of water into this pot?" (If possible, supply the water and jars at the station, so the children can physically do the task).

Fractions tasks - each station based around a different theme- Example: different ways to express '1/2' (50%, 0.5, 2/4, 3/6). Include props, such as blocks or coloured paper cut into a number of fractions.

**•Humanities**: Historical events. These could be read-and-comprehend tasks- Example: "In which year did Captain Cook discover Australia? Describe his landing."

**•Creative task**: "Imagine you are living in Antarctica, draw some of the clothes you would need to wear to keep warm."

### Options

•At the end of the lesson, the children can come together and the teacher can lead a discussion of their work; or groups can exchange work for correction.

### Equipment/preparation required

✓Activities for each station (copies/laminated cards of reading passages, math problems, props for problem-solving activities etc)

✓Pens/pencils and paper/workbooks (for children to record answers at each station)

✓ *(optional) *One clipboard per group/per child

✓ *(optional) *Worksheets for each station (if these are to be completed)

## Appendix 2

'Road trip/Stations around the room' standing lesson strategy

### Aims

To practice spelling or simple number patterns verbally while standing and passing a bean-bag.

### Outline

The children are asked to stand and the teacher nominates a number series or a set of words, relevant to the current lesson. The teacher gives a bean-bag to one student, who states the first number in the pattern or letter in the word. That child then throws the bean-bag to another child, who says the next number/letter then passes the bean-bag on, and so on.

All children remain standing throughout the game, but once a child has passed the bean-bag, they must change their posture/position to signify that they have had their turn.

**Table:** Teachers can specify

Subject of game (examples):	The actions performed by children before/after their turns (examples):
• *Spell "..." (word from the current spelling list or story being read)* • *Multiples of... (during a maths lesson)* • *Count up to 100 by twos/fives*	• *Everyone stand up, when you've had your turn, stand on one leg* • *Everyone stand up, after your turn stand on one leg/bend down and touch your toes* • *Everyone stand with their arms out to the sides, after your turn, place hands on head* • *Everyone jog on the spot, after you've had your turn, start hopping*

### Equipment/preparation required

✓Bean-bag (provided) or newspaper balls to throw

*✓ (optional) *Spelling-list on the board

## Pre-publication history

The pre-publication history for this paper can be accessed here:

http://www.biomedcentral.com/1471-2458/11/759/prepub
